# Unwillingness to engage in behaviors that protect against COVID-19: the role of conspiracy beliefs, trust, and endorsement of complementary and alternative medicine

**DOI:** 10.1186/s12889-021-10643-w

**Published:** 2021-04-08

**Authors:** Anna Soveri, Linda C. Karlsson, Jan Antfolk, Mikael Lindfelt, Stephan Lewandowsky

**Affiliations:** 1 Institute of Clinical Medicine, University of Turku, Turku, Finland; 2 Department of Psychology, Åbo Akademi University, Turku, Finland; 3 Department of Theological Ethics, Åbo Akademi University, Turku, Finland; 4 School of Psychological Science, University of Bristol, Bristol, UK; 5 School of Psychological Science, University of Western Australia, Perth, Australia

**Keywords:** COVID-19, Vaccine attitudes, Non-pharmaceutical interventions, NPI, Conspiracy, Complementary and alternative medicine, CAM, Trust

## Abstract

**Background:**

We investigated if people’s response to the official recommendations during the COVID-19 pandemic is associated with conspiracy beliefs related to COVID-19, a distrust in the sources providing information on COVID-19, and an endorsement of complementary and alternative medicine (CAM).

**Methods:**

The sample consisted of 1325 Finnish adults who filled out an online survey marketed on Facebook. Structural regression analysis was used to investigate whether: 1) conspiracy beliefs, a distrust in information sources, and endorsement of CAM predict people’s response to the non-pharmaceutical interventions (NPIs) implemented by the government during the COVID-19 pandemic, and 2) conspiracy beliefs, a distrust in information sources, and endorsement of CAM are related to people’s willingness to take a COVID-19 vaccine.

**Results:**

Individuals with more conspiracy beliefs and a lower trust in information sources were less likely to have a positive response to the NPIs. Individuals with less trust in information sources and more endorsement of CAM were more unwilling to take a COVID-19 vaccine. Distrust in information sources was the strongest and most consistent predictor in all models. Our analyses also revealed that some of the people who respond negatively to the NPIs also have a lower likelihood to take the vaccine. This association was partly related to a lower trust in information sources.

**Conclusions:**

Distrusting the establishment to provide accurate information, believing in conspiracy theories, and endorsing treatments and substances that are not part of conventional medicine, are all associated with a more negative response to the official guidelines during COVID-19. How people respond to the guidelines, however, is more strongly and consistently related to the degree of trust they feel in the information sources, than to their tendency to hold conspiracy beliefs or endorse CAM. These findings highlight the need for governments and health authorities to create communication strategies that build public trust.

**Supplementary Information:**

The online version contains supplementary material available at 10.1186/s12889-021-10643-w.

## Background

As of early 2020, the world has been dealing with a global health crisis caused by the COVID-19 pandemic. In the absence of any effective treatments or vaccines for the disease, governments worldwide implemented a wide range of non-pharmaceutical interventions (NPIs), such as social distancing, school closures, remote working, restrictions concerning public gatherings, quarantines, hand-washing and the use of masks to slow transmission of the disease [1]. Those measures (e.g., school closures and stay-at-home-orders) have been shown to be effective in reducing the number of infections [2, 3], but that success has been accompanied by substantial economic, social [4] and psychological costs (for a review, see [5]). To ease the burden on society, a great effort worldwide has been put into developing and getting access to a vaccine [6, 7]. A year after the first reported cases of COVID-19, countries around the world have started vaccinations [8].

What characterizes NPIs and immunization programs is that their success is - to a great degree - dependent on the public’s acceptance and compliance. It is worrying, therefore, that a large number of studies suggest that not all individuals comply with government-implemented NPIs during the COVID-19 pandemic [9–19]. When it comes to a vaccine against COVID-19, recent studies alarmingly show that although most people would take the vaccine, many individuals report that they feel hesitant towards it or would not get vaccinated [15, 18–22]. If the COVID-19 vaccine uptake is insufficient, thus preventing or delaying herd immunity, NPIs will continue to play an important role in managing the spread of the disease [23].

The willingness of the public to comply with the NPIs and to take the COVID-19 vaccine is essential in how the pandemic plays out, and thus it is of great importance to understand the motives behind non-compliance. The present study focuses on the role of three factors: conspiracy beliefs, a distrust in the institutions providing health information, and an endorsement of complementary and alternative medicine (CAM). Conspiracy beliefs are known to be involved in nearly all forms of science denial (e.g., [24]; for a summary, see [25, 26]), and COVID-19 is no exception [27, 28]. Several studies have shown that conspiracy beliefs are a particularly strong predictor of the rejection of vaccinations [24, 29], including the rejection of COVID-19 vaccines [30]. Conspiracy beliefs are also related to the other factors. Individuals with more conspiracy beliefs have lower trust in science or political and medical authorities [11, 15, 17, 18, 31, 32] and a higher likelihood to turn to CAM [17, 19, 31, 33], possibly due to a distrust in the biomedical system [34]. People who endorse CAM have less trust in medical authorities [35].

What these three factors — conspiracy beliefs, low trust in authorities, and endorsement of CAM— may have in common is that they imply a tendency to question the appropriateness of the recommendations given by governments and health authorities. In the context of COVID-19, this may lead to non-compliance with NPIs and vaccination recommendations. In fact, recent studies suggest that people’s unwillingness to engage in health-protective behavior is associated with more conspiracy beliefs [11, 14–19, 27, 31, 33, 36–38], a distrust in the establishment [10, 17, 22], and an endorsement of CAM [33].

### Conspiracy beliefs

A particular challenge for health authorities in managing the public’s response to NPIs and the vaccine against COVID-19 is countering COVID-19 related misinformation and conspiracy beliefs [39]. Holding conspiracy beliefs typically implies believing that a secretive group of people with malicious intentions are behind a particular event [40]. Conspiracy beliefs are common during public health crises and may influence people not to take preventive actions [41]. The COVID-19 pandemic provides a fertile breeding ground for conspiracy theories, because it is a complex event that causes fear and is difficult to understand [42]. Indeed, studies show that conspiracy theories about COVID-19 are common [11, 15, 27] and relate, for example, to the origin and spread of the virus, the reasons behind the NPIs or the development of a vaccine [43, 44]. Alarmingly, individuals who believe in COVID-19 conspiracy theories are more likely to have negative attitudes to a government’s response to the pandemic [31, 36], not adhere to NPIs, such as handwashing or social distancing [11, 14–19, 27, 31, 37] and reject a future vaccination against COVID-19 [15, 18, 19, 30, 33, 37, 38]. These results are in line with previous studies investigating the relationship between conspiracy beliefs and negative attitudes to other vaccines [24, 45, 46]. However, not all studies find an effect between conspiracy beliefs and people’s compliance with the official guidelines during COVID-19 [13, 47].

### Trust

The COVID-19 pandemic is a “rapidly evolving event characterized by scientific uncertainty” [48]. This uncertainty and constantly evolving science on COVID-19, has made health communication during the pandemic difficult [49], as it has resulted not only in a massive flow of health information, but also in rapidly changing information, mixed messages and inconsistencies in recommendations. The possible consequences of this on the public’s trust in health communicators and policy makers is a matter of concern [48] because low trust has been related to a smaller likelihood of following recommendations given by health authorities during previous outbreaks of infectious diseases [50, 51]. The importance of trust has also been emphasized in studies on COVID-19, as stronger public trust has been related to lower COVID-19 mortality [52], a greater reduction in human mobility during lockdown [53], a higher likelihood of individuals complying with the NPIs [10, 17, 18], and more willingness to take a COVID-19 vaccine [18, 22]. A lack of trust in medical authorities is an important antecedent of antivaccination attitudes [35, 54–58] and antivaccination behavior [35] also when it comes to other vaccines.

### CAM endorsement

CAM is an umbrella term for a wide range of treatments and substances that fall outside the conventional care recommended in a country. By definition, CAM can be used either in addition to, or instead of, conventional treatments [59]. Approximately 30% of adults living in the U.S. [60] and 10–40% in Europe [61] use CAM. Studies show that individuals who use CAM, or have positive attitudes to CAM, have a lower likelihood of complying with conventional treatments, such as vaccinations [35, 62–67]. It has been suggested that the relationship between positive attitudes to CAM and negative attitudes to vaccines is due to an underlying view on health that is not evidence-based and an unwillingness to adhere to conventional medicine [62–64]. Among those who endorse CAM, CAM is also considered a natural, non-toxic way to strengthen the immune system, while vaccines are perceived as harmful [63]. Furthermore, both CAM use and antivaccination attitudes have been shown to be related to lower trust in medical authorities [35]. As regards COVID-19, recent studies suggest that people with more positive attitudes to CAM are more unwilling to accept a COVID-19 vaccine [33] and to follow the official COVID-19 guidelines [17].

### The present study

To shed light on the reasons behind people’s unwillingness to adhere to official recommendations during the COVID-19 pandemic, we tested hypotheses derived from previous research suggesting that stronger COVID-19 related conspiracy beliefs, more endorsement of CAM, and a lower trust in the sources providing information on COVID-19, are related to more negative responses (compliance with the NPIs and emotional response to the NPIs) towards the NPIs implemented by the government during the pandemic. We also tested the hypotheses that COVID-19 conspiracy beliefs, a lack of trust in information sources and more positive attitudes to CAM are related to an unwillingness to take a vaccine against COVID-19. Finally, we explored whether people’s responses to the NPIs are related to their willingness to take the COVID-19 vaccine, and whether this association can be explained by conspiracy beliefs, a lower trust in the information sources, and more endorsement of CAM.

## Methods

### Participants and procedure

The respondents were adults living in Finland recruited through a Facebook post. The post was marketed for 2 weeks during the first peak of COVID-19, between the 3rd and 17th of April 2020. The post reached 97,408 Facebook users and was viewed by 3305 (3.4%) individuals. Of these, 2233 (67.6%) filled out at least parts of the online questionnaire. The present study included only those 1325 individuals who had responded correctly to an attention check question and reached the end of the questionnaire (i.e., responded to questions on the last page of the survey). Of those individuals, 1023 reported their age (*M* = 41.71, *SD* = 13.11, range = 18–100). See Table 1 for more information on the respondents. The sample is the same as in a previous study [21], but the analyses reported here are new.

**Table 1:** Sample descriptives

Variable	*n*	*%*
Age^a^		
18–29	101	9.87
30–39	185	18.08
40–49	255	24.93
50–59	275	26.88
60–69	166	16.23
70+	41	4.01
Gender^b^		
Female	1054	79.61
Male	253	19.11
Other	7	0.53
Did not want to report	10	0.76
Education		
Primary school	117	8.84
Vocational education	505	38.14
High school	168	12.69
Bachelor’s degree	277	20.92
Master’s degree	191	14.43
Doctoral degree	27	2.04
Other	39	2.95
Language		
Finnish	1181	89.20
Swedish	143	10.80

^a^Information on 302 (22.8%) individuals missing

^b^Information on 1 (0.1%) individual missing

Before filling out the questionnaire, the respondents were informed that participation in the study was anonymous and voluntary, and that they could withdraw at any time. All participants gave their written informed consent by clicking “I agree to participate in the study”, before proceeding to the questionnaire.

### Measures

The survey was administered in either Finnish or Swedish, depending on the preference of the participant. The measures included in the study are described below and in Table 2. Except for the items measuring CAM endorsement and the emotional response to the NPIs, all items in the questionnaire have been developed for the present study. See Additional file 1 for the questionnaire in English.

**Table 2:** Survey questions and labels

Construct	Question	Label
NPI compliance^a^	I am motivated to behave in accordance with the authorities’ recommendations.	Behav_Motivated
	I have been ready to make changes in my behavior in order not to get infected with the coronavirus.	Behav_Self
	I have been ready to make changes in my behavior in order not to spread the coronavirus.	Behav_Other
	I see no reason to change my behavior despite the corona pandemic. (REV)	Behav_Reason
NPI emotional response^b^	Item 1	StateR_Freedom
	Item 2	StateR_Frustrate
	Item 3	StateR_Irritate
	Item 4	StateR_Upset
Vaccination intentions^c^	Imagine a hypothetical scenario where the authorities recommend a new vaccine against COVID-19 free of charge. How likely do you consider it to be that you would accept such a vaccine?	CovVacc_Recom
Conspiracy beliefs^a^	A hidden organization is behind the spread of the coronavirus.	Consp_Hidden
	Pharmaceutical companies are behind the spread of the coronavirus.	Consp_Pharma
	Financial interests lie behind the spread of the coronavirus.	Consp_Econ
	The coronavirus pandemic is made up.	Consp_Madeup
Trust^a^	I trust what medical doctors say about the coronavirus pandemic.	Trust_Doctors
	I trust what scientists say about the coronavirus pandemic.	Trust_Researchers
	I trust what media (e.g., YLE) reports about the coronavirus pandemic.	Trust_Media
	I trust the information provided by authorities (e.g., THL) about the coronavirus pandemic.	Trust_Authority
CAM endorsement^a^	Item 1 (REV)	CAM_Danger
	Item 2	CAM_Cure
	Item 3 (REV)	CAM_Ineffect
	Item 4	CAM_Saves
	Item 5	CAM_Superior

*REV* Reverse-scored item, *YLE* Finland’s national public broadcasting company, *THL* Finnish Institute for Health and Welfare

^a^Scale 1 (*completely disagree*) to 5 (*completely agree*)

^b^Scale 1 (*not at all*) to 5 (*very much*)

^c^Scale 1 (*very unlikely*) to 5 (*very likely*)

#### Response to the NPIs during COVID-19

We measured two aspects of people’s response to the NPIs during the COVID-19 pandemic: compliance with the NPIs and emotional response to the NPIs. Compliance with the NPIs was measured with four statements (e.g., “I am motivated to behave in accordance with the authorities’ recommendations”). The respondents indicated to what extent they agreed on a scale from 1 (*completely disagree*) to 5 (*completely agree*). Due to the evolving circumstances related to the NPIs implemented by the Finnish government at the time of data collection, we decided to measure how motivated people were to comply with NPIs in general, instead of measuring their compliance with specific NPIs.

The emotional response to the NPIs was measured using Finnish and Swedish translations of the Experience of Reactance subscale in the Salzburger State Reactance Scale [68]. The subscale consisted of four questions probing the respondents’ emotional reactions to the government’s restrictions during the COVID-19 pandemic. The answers were given on a 1 (*not at all*) to 5 (*very much*) scale.

#### COVID-19 vaccination intentions

To gain information on the respondents’ willingness to take a prospective vaccine against COVID-19, the survey included the following question: “Imagine a hypothetical scenario where the authorities recommend a new vaccine against COVID-19 free of charge. How likely do you consider it to be that you would accept such a vaccine?” The respondents answered this question on a scale from 1 (*very unlikely*) to 5 (*very likely*).

#### Belief in conspiracies

Four statements were created to assess to what degree the respondents believed in COVID-19 related conspiracy theories (e.g., “A hidden organization is behind the spread of the coronavirus”). The answers were given on a scale from 1 (*completely disagree*) to 5 (*completely agree*). Because the data collection was conducted in the early stages of the pandemic, all conspiracy items refer to general, unspecified outgroups instead of specific conspiracy theories that may have been unfamiliar to the respondents at that time.

#### Trust in information sources

Trust in the sources providing information on COVID-19 was measured using four statements, each of them querying a specific source of information. The four sources were: the government, health authorities, scientists and news media (e.g., “I trust the information provided by authorities [e.g., THL] about the coronavirus pandemic”). The respondents gave their answers on a scale from 1 (*completely disagree*) to 5 (*completely agree*).

#### CAM endorsement

To measure CAM endorsement, we used Finnish and Swedish translations of the five items used by Lewandowsky, Woike, and Oberauer [67]. Two of the items in that scale were taken from Hyland, Lewith, and Westoby [69]. In the study by Lewandowsky et al. [67], higher scores indicated a rejection of CAM. In this study, we used the opposite polarization of the construct, so that higher scores indicated CAM endorsement. The respondents answered the statements on a scale from 1 (*completely disagree*) to 5 (*completely agree*).

### Statistical analysis

We used structural regression (SR) analysis using the package *lavaan* [70] in *R* version 3.5.1 [71]. Compliance with NPIs during COVID-19, emotional response to NPIs during COVID-19, COVID-19 conspiracy beliefs, trust in COVID-19 information sources, and CAM endorsement, were represented by latent factors. We specified an SR model with three outcome measures: NPI compliance, NPI emotional response, and vaccination intentions. The outcomes were regressed on the factors Conspiracy beliefs, Trust, and CAM endorsement. Then we investigated whether Conspiracy beliefs, Trust, and CAM endorsement explained the possible associations between people’s response to the NPIs (NPI compliance and NPI emotional response) and their vaccination intentions. This was done by exploring whether the disturbance correlations (i.e., the correlations between the proportion of the variances that are not explained by the predictors) between the outcome measures were weaker than their zero-order correlations. We applied WLSMV estimation due to ordinal indicators and skewed distributions. We applied pairwise deletion of missing values.

## Results

**Fig. 1:**
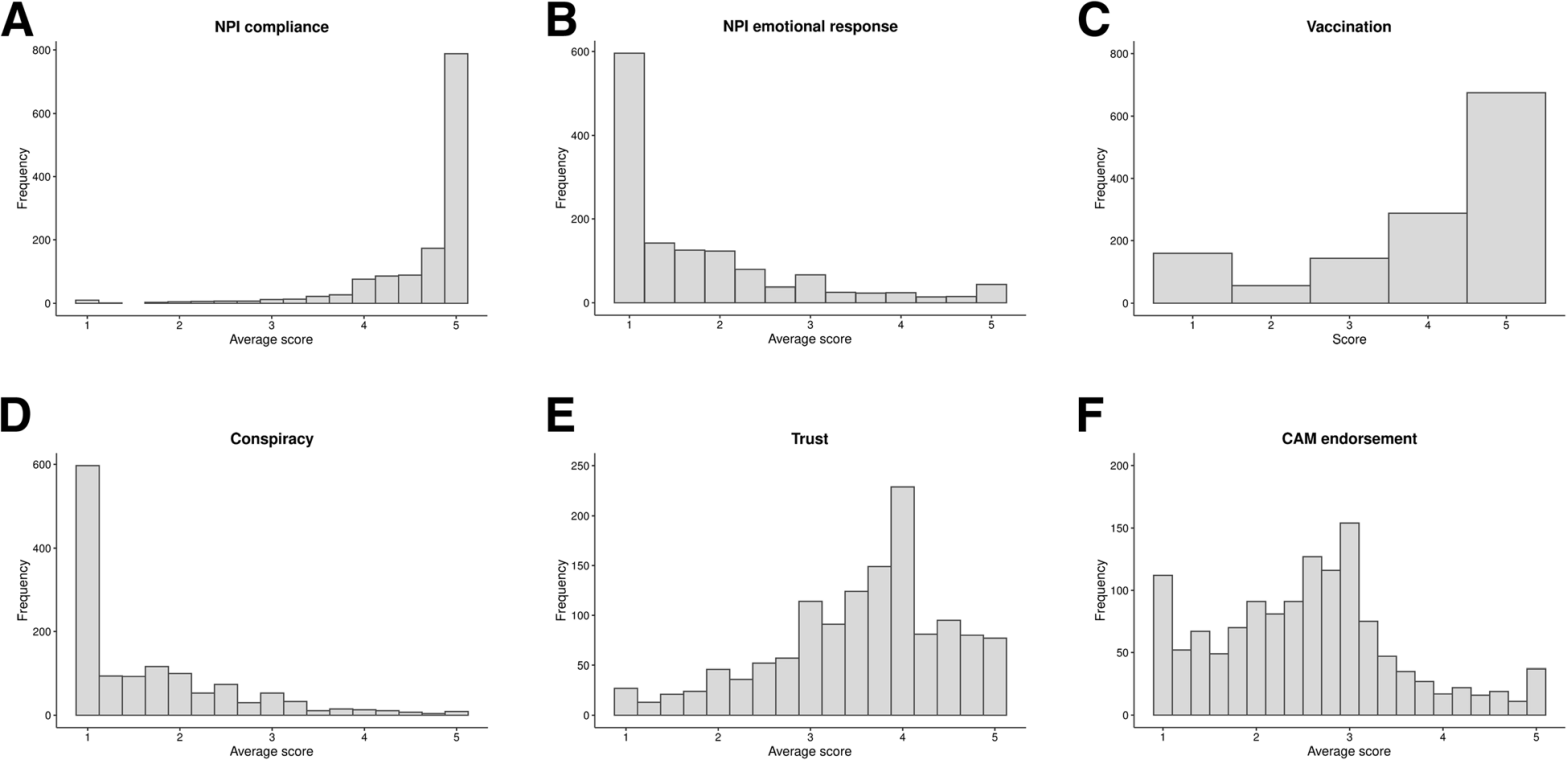
Distribution of Responses on the Measures of NPI Compliance, Vaccination Intention, NPI emotional response, Conspiracy Beliefs, Trust, and CAM Endorsement

The responses to all statements are presented in Table 3 and average scores for the individuals on each factor are presented in Fig. 1. Zero-order correlations are presented in Table 4. In the following, *p*-values lower than .001 are considered statistically significant.

**Table 3:** Responses to the questions on NPI compliance, NPI emotional response, vaccination intentions, conspiracy beliefs, trust, and CAM endorsement

	1		2		3		4		5	
	*n*	*%*	*n*	*%*	*n*	*%*	*n*	*%*	*n*	*%*
NPI compliance^a^										
Behav_Motivated	21	1.58	22	1.66	65	4.91	306	23.09	911	68.75
Behav_Self	19	1.44	12	0.91	54	4.08	195	14.73	1044	78.85
Behav_Other	14	1.06	10	0.76	41	3.10	173	13.07	1086	82.02
Behav_Reason (REV)	1066	80.51	133	10.05	54	4.08	34	2.57	37	2.79
NPI emotional response^b^										
StateR_Freedom^c^	308	23.32	341	25.81	287	21.73	245	18.55	140	10.60
StateR_Frustrated	763	57.89	269	20.41	129	9.79	80	6.07	77	5.84
StateR_Annoyed	767	58.15	284	21.53	135	10.24	61	4.62	72	5.46
StateR_Upset	812	61.47	251	19.00	133	10.07	57	4.31	68	5.15
Vaccination intentions^d^	159	12.05	55	4.17	143	10.84	288	21.83	674	51.10
Conspiracy beliefs^a^										
Consp_Hidden	709	53.55	213	16.09	252	19.03	91	6.87	59	4.46
Consp_Pharma	854	64.75	216	16.38	177	13.42	44	3.34	28	2.12
Consp_Econ	745	56.40	232	17.56	209	15.82	89	6.74	46	3.48
Consp_Madeup	1091	82.53	87	6.58	97	7.34	23	1.74	24	1.82
Trust^a^										
Trust_Doctors	68	5.14	118	8.91	284	21.45	542	40.94	312	23.56
Trust_Scientists	43	3.25	76	5.75	258	19.52	591	44.70	354	26.78
Trust_Media	112	8.47	170	12.86	409	30.94	503	38.05	128	9.68
Trust_Authorities	147	11.14	185	14.02	301	22.80	446	33.79	241	18.26
CAM endorsement^a^										
CAM_Danger (REV)	167	12.61	144	10.88	373	28.17	297	22.43	343	25.91
CAM_Cure	310	23.47	291	22.03	471	35.65	157	11.88	92	6.96
CAM_Ineffect (REV)	131	9.92	182	13.79	442	33.48	221	16.74	344	26.06
CAM_Saves	323	24.43	301	22.77	481	36.38	118	8.93	99	7.49
CAM_Superior	380	28.77	304	23.01	451	34.14	102	7.72	84	6.36

*REV* reverse-score items

^a^Scale 1 (*completely disagree*) to 5 (*completely agree*)

^b^Scale 1 (*not at all*) to 5 (*very much*)

^c^Item not included in the final NPI emotional response factor

^d^Scale 1 (*very unlikely*) to 5 (*very likely*)

**Table 4:** Zero-order correlations between all measures

Variable	1	2	3	4	5	6
1. Conspiracy beliefs	–					
2. Trust	−.57	–				
3. CAM endorsement	.59	−.49	–			
4. NPI compliance	−.49	.55	−.44	–		
5. NPI emotional response	.44	−.48	.31	−.62	–	
6. Vaccination intentions	−.45	.58	−.50	.48	−.32	–

*Note.* All *p*s < .001

### Measurement models

Factor fit statistics are presented in Table 5. The fit of the factor NPI emotional response including the four items was good, with the exception of a high RMSEA statistic (χ^2^[2] = 60.21, CFI = .998, TLI  =   .994, RMSEA =   .148; 90% CI[.117, .182], SRMR = .039). One of the items, StateR_Freedom, loaded weakly on the factor (standardized loading .34), whereas the standardized loadings of the remaining three items were all < .87. Therefore, we removed StateR_Freedom from the factor. Fit statistics for the final factor could not be estimated as the model is fully saturated (*df* = 0). The RMSEA statistic was high also for the factor Trust (χ^2^[2] = 29.95, CFI = .998, TLI  =   .995, RMSEA =  .103; 90% CI[.072, .137], SRMR = .025). Because the suggested residual correlations were negative, and thus not in accordance with the a priori assumption, we decided not to include them in the model. This was done also to avoid the risk of overfit. However all other fit indeces were good and factor loadings were high (standardized loadings all > .77).

**Table 5:** Fit statistics of the factors

Factor	*χ*^2^(*df*)	CFI	TLI	RMSEA[90% CI]	SRMR
NPI compliance	0.82 (2)	1.00	1.00	.000[.000, .042]	.006
Conspiracy beliefs	5.35 (2)	1.00	.999	.036[.000, .074]	.009
Trust	29.95 (2)	.998	.995	.103[.072, .137]	.025
CAM endorsement^a^	42.71 (4)	.997	.993	.085[.063, .110]	.017

^a^Residual correlation between the two reversed items included (*r* = .09, *p* < .001)

### SR analysis

**Fig. 2:**
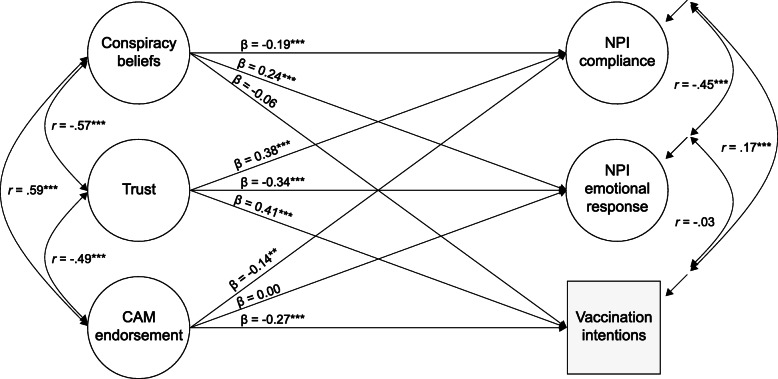
Standardized estimates, correlations between predictors, and disturbance correlations between outcomes. Factor indicators, loadings, and variances are not shown in the figure

The SR model fitted the data well (*χ*^2^[174] = 6334.53, CFI = .990, TLI  =   .988, RMSEA =   .045; 90% CI[.041, .048], SRMR = .042). Conspiracy beliefs and Trust were statistically significant predictors of both measures of people’s response to the NPIs: NPI compliance and NPI emotional response (Table 6 and Fig. 2), indicating that a more negative response to the NPIs was related to more conspiracy beliefs and lower trust in COVID-19 information sources. COVID-19 vaccination intentions were significantly predicted by Trust and CAM endorsement, so that lower trust and more positive attitudes to CAM were associated with a smaller likelihood of accepting the vaccine. The model explained 36% (*R*^*2*^ = 0.363) of the variance of NPI compliance, 27% (*R*^*2*^ = 0.268) of NPI emotional response, and 40% (*R*^*2*^ = 0.398) of COVID-19 vaccination intention.

**Table 6:** Predictors of compliance with NPIs and COVID-19 vaccination intentions

Variable	Standardized estimates			
	*β*	95% CI	*z*	*p*
NPI compliance				
Conspiracy beliefs	−.19	[−.29, −.10]	3.92	< .001
Trust	.38	[.30, .45]	9.75	< .001
CAM endorsement	−.14	[−.23, −.05]	3.03	.002
NPI emotional response				
Conspiracy beliefs	.24	[.15, .33]	5.29	< .001
Trust	−.34	[−.41, −.27]	9.36	< .001
CAM endorsement	.00	[−.08, .08]	0.11	.914
Vaccination intentions				
Conspiracy beliefs	−.06	[−.13, .01]	1.61	.107
Trust	.41	[.36, .47]	14.95	< .001
CAM endorsement	−.27	[−.32, −.21]	9.01	< .001

The disturbance correlation between NPI compliance and NPI emotional response was *r* = −.45; 95% CI[−.52, −.38], whereas the zero-order correlation was *r* = −.62; 95% CI[−.67, −.56]. The fact that the disturbance correlation was lower than the zero-order correlation, with non-overlapping confidence intervals, suggests that Conspiracy beliefs and Trust explain some of the association between NPI compliance and NPI emotional response. For the outcomes NPI compliance and vaccination intentions, and NPI emotional response and vaccination intentions, the disturbance correlations were *r* = .17; 95% CI[.11, .23] and *r* = −.03; 95% CI[−.09, .03], respectively, and the zero-order correlations were *r* = .48; 95% CI[.42, .54] and *r* = −.32; 95% CI[−.38, −.27] respectively. These results indicate that Trust explained some of the association between the response to the NPIs and vaccination intentions.

## Discussion

How the COVID-19 pandemic develops is - to a great degree - dependent on the public’s compliance with government measures taken to manage the spread of the disease. Alarmingly, recent reports suggest that even though a majority of the public adheres to the official recommendations, non-compliance does exist as well [9–19]. Additionally, some individuals are unwilling to take a vaccine against COVID-19 [15, 18–22]. The results from the present study conducted during the first peak of COVID-19, support those findings. They show that on average, 93% of the respondents had been completely or somewhat willing (78 and 15% respectively) to comply with the NPIs implemented during the COVID-19 pandemic, and on average, 10% felt frustrated, annoyed, or upset because of the NPIs. Furthermore, 73% considered it very or somewhat likely (51 and 22% respectively) that they would take a vaccine against COVID-19, if the authorities recommended it and if it were free of charge. These percentages roughly correspond to the results from previous studies conducted in other countries [15, 18, 20, 22].

The main aim of the present study was to investigate why some individuals have a negative response to official recommendations (NPIs and vaccine) during the COVID-19 pandemic. The results showed that those people who are unwilling to comply with the NPIs, or who react with negative emotions towards the NPIs, have more conspiracy beliefs and a lower trust in the sources providing information on COVID-19. Trust was important also in people’s willingness to take the COVID-19 vaccine, as individuals who are reluctant to get vaccinated, have a lower trust in the COVID-19 information sources. They also have a more positive attitude to CAM.

The results supported previous studies showing that lower trust is related to less compliance with the NPIs [10, 17] and a lower likelihood of wanting to take the COVID-19 vaccine [18, 22]. Trust in the sources providing information on COVID-19 was the strongest predictor of all three outcomes, explaining approximately 12–14% of the variance in people’s response to the NPIs and 20% in people’s willingness to take the vaccine. Of the four listed sources of information (i.e., medical doctors, scientists, news media, authorities), only approximately half of the respondents completely or somewhat trusted the authorities and the media in providing accurate information on COVID-19. Medical doctors and scientists were perceived as being more trustworthy, as 65% of the respondents completely or somewhat trusted doctors, and 71% completely or somewhat trusted scientists.

In line with previous research [11, 14–19, 27, 31, 36, 37], the results indicated that people who have stronger COVID-19 conspiracy beliefs have a more negative response towards the NPIs. However, the hypothesis that people who have stronger COVID-19 conspiracy beliefs are less willing to take a vaccine against COVID-19 [15, 18, 19, 33, 37, 38], was not supported. The zero-order correlation between conspiracy beliefs and COVID-19 vaccination intentions was medium strong (*r* = −.45), indicating that more conspiracy beliefs are related to more unwillingness to take a COVID-19 vaccine. When controlling for the other predictors in the model, however, the unique effect of conspiracy beliefs did not reach statistical significance. On average 8% believed that the conspiracy theories are completely or somewhat true (3 and 5%, respectively). Of the four listed conspiracy theories, beliefs that a hidden organization or financial interests lie behind the spread of COVID-19, received the most support. The average proportion of people who - to some degree - believe in the conspiracy theories, is lower than in previous studies. For example, in two studies investigating COVID-19 related conspiracy beliefs in the UK [15, 27], the average percentage of people believing in the COVID-19 related conspiracy theories was almost twice as high as in the present study. One possible reason for this discrepancy relates to which conspiracy beliefs were included in the questionnaires. However, for the same conspiracy theories, endorsement was also higher in the two studies conducted in the UK [15, 27]. For example, the statement that the COVID-19 pandemic was a hoax was supported by approximately 7% in the Allington et al. [27] study, compared to 4% in the present study, and items stating that financial gains are behind the spread of the virus were supported by approximately 12–14% in the study by Freeman et al. [15] and 10% in the present study.

The finding that people with more positive attitudes to CAM were less willing to take a COVID-19 vaccine was in accordance with previous research [33], supporting the notion that for some people negative attitudes to vaccines may be due to an unwillingness to adhere to conventional medicine [62–64]. The correlations between endorsement of CAM and the two outcomes measuring people’s response to the NPIs were statistically significant and moderate, indicating that more endorsement of CAM was related to a more negative response to the NPIs. There was, however no statistically significant unique effect of CAM endorsement when controlling for the other predictors in the model. Similar findings were reported in the previous study investigating the role of conspiracy beliefs, trust, and CAM beliefs in people’s compliance with NPIs [17]. That study demonstrated a statistically significant correlation between CAM beliefs and NPI compliance, but no mediating effect of CAM beliefs when studying the effects of conspiracy beliefs on NPI compliance. Of the participants in the present study, approximately 47% completely or somewhat endorsed CAM (26 and 21%, respectively). These numbers are higher than in the previous study [67] where the same set of statements were used to measure CAM attitudes in the UK. In that study, approximately 35% had positive attitudes to CAM to some degree.

The results from the present study also showed that COVID-19 related conspiracy beliefs, distrust in the sources of COVID-19 information, and endorsement of CAM, are moderately to strongly correlated, supporting previous studies [18, 19, 31, 33, 35]. People with lower trust in the establishment giving accurate information had stronger conspiracy beliefs, and a more positive attitude to CAM. The three predictors together explained approximately 36% of how willing people were to comply with the NPIs, 27% of how frustrated, annoyed or upset people felt about the NPIs, and 40% of how willing they were to take a COVID-19 vaccine. Furthermore, the results indicated that some of the people who respond negatively to the NPIs also have more unwillingness to take the vaccine. This association was related to the predictors in the model, in particular to a lower trust in the establishment providing accurate information.

The present results thus show that the level of trust people feel towards political authorities, health authorities, scientists, and the media, is consistently related to what degree they are willing to adhere to the official guidelines during COVID-19. This underlines the importance of taking action towards building public trust in order to ensure acceptance and compliance with the NPIs and the vaccine. A key factor in building trust during a pandemic is transparent communication [48, 71]. Correcting conspiracy theories is challenging, particularly among strong believers of conspiracy theories [72]. One explanation is that evidence *against* a theory may be interpreted as evidence *supporting* the theory, because the people who are providing the evidence are seen as part of the conspiracy [73]. However, some suggestions on how to tackle conspiracy theories do exist, such as providing anti-conspiracy (i.e., accurate scientific information) information prior to the conspiracy theories becoming established [74], or approaching the issue by treating the possible underlying motives, such as feelings of powerlessness [42, 72], distrust, and alienation [42].

### Limitations

In the present study, we assumed that compliance with the official recommendations (NPIs and vaccine) are underpinned by conspiracy beliefs, CAM endorsement, and trust in information sources. The cross-sectional design does not allow us to draw causal inferences. Based on previous literature, however, it seems plausible to assume that conspiracy beliefs, CAM endorsement, and trust explains compliance with the NPIs and not the other way around. Additionally, as a COVID-19 vaccine was not yet available at the time of data collection, it seems unlikely that the intentions to take the vaccine would influence people’s beliefs in conspiracy theories, attitudes to CAM, or trust in the information sources.

The fact that the data collection was conducted over Facebook may have influenced the generalizability of the results. This is because it may have led to sampling bias due to self-selection. It is possible that individuals with certain characteristics, for example very negative or very positive attitudes to the COVID-19 official guidelines or a COVID-19 vaccine, may have been more interested in participating in the study. The histograms in Fig. 1, however, reveal that for both NPI compliance and vaccination intentions, our results are approximately in line with previous studies, suggesting that in this regard, the sample in the present study did not clearly deviate from other samples. Important to note, however, is that the gender distribution in the present study was very uneven, with men constituting only 1/5 of the total sample. Furthermore, the age distribution indicates that most respondents were between 40 and 60 years of age. These asymmetries are important to keep in mind when generalizing the results.

A general issue with self-reported survey data, is the risk of response bias, caused for example by respondents answering the questions in a socially desirable way. This may affect the validity of the results. However, a recent study suggests that self-reports of NPI compliance during COVID-19 do not seem to suffer from social desirability bias [75]. To decrease the risk of social desirability bias in the present study, the respondents were informed that participation in the study was completely anonymous. To work against response bias further, the survey included an attention check, where the participants were asked to select a specific number on a scale. Only those participants answering the attention check correctly were included in the study. Furthermore, NPI compliance and CAM endorsement included reverse-scored items.

The questionnaires assessing conspiracy beliefs, trust in information sources, NPI compliance, and vaccination intentions have not been validated in other samples. For all constructs other than vaccination intentions, however, factor analysis was used to assess the factor loadings of the questions on the constructs and to handle measurement error. Factor analysis was not performed on vaccination intentions because the survey included only one question about people’s intentions to take a COVID-19 vaccine. The reason for this was that we wanted to obtain information specifically on people’s willingness to take a vaccine in a situation where the vaccine is recommended by the authorities and free of charge.

## Conclusions

Taken together, the results show that people who have a lower trust in the establishment in providing accurate information on COVID-19 and more conspiracy beliefs have a more negative response towards the NPIs. People who have a lower trust in the establishment and endorse CAM, are more unwilling to take the COVID-19 vaccine. The degree of trust people feel towards the establishment thus plays an important role in how they respond to the official recommendations during the COVID-19 pandemic. These findings underline the importance of taking action towards building public trust, in order to ensure compliance with the NPIs and acceptance of the COVID-19 vaccine.

## Supplementary Information

**Additional file 1.** Questionnaire in English

## Data Availability

The datasets used and analysed during the current study are available in the OSF repository at https://osf.io/8djw2/.

## Abbreviations

NPI: Non-pharmaceutical interventions
CAM: Complementary and alternative medicine
