# Supplementary material for: Unwillingness to engage in behaviors that protect against COVID-19: the role of conspiracy beliefs, trust, and endorsement of complementary and alternative medicine
Source: BMC Public Health. 2021 Apr 8;21:684. doi: 10.1186/s12889-021-10643-w (PMC8027965; doi:10.1186/s12889-021-10643-w)
Supplement: Supplementary file 1 — Additional file 1. Questionnaire in English [file 12889_2021_10643_MOESM1_ESM.docx]

**Questionnaire in English**

In the beginning of the year, a novel infectious disease (COVID-19) started spreading in China. The virus has now spread to almost all parts of the world. On March 11th, the World Health Organization (WHO) declared the outbreak a pandemic. The virus exists also in Finland.

The questions below concern this coronavirus pandemic.

TRUST IN INSTITUTIONS

1 (*completely disagree*) to 5 (*completely agree*)

1. I trust what medical doctors say about the coronavirus pandemic.
2. I trust what scientists say about the coronavirus pandemic.
3. I trust what media (e.g., YLE) reports about the coronavirus pandemic.
4. I trust the information provided by authorities (e.g., THL) about the coronavirus pandemic.

VACCINATION INTENTIONS

1 [*very unlikely*] till 5[*very likely*]

1. Imagine a hypothetical scenario where the authorities recommend a new vaccine against COVID-19 free of charge. How likely do you consider it to be that you would accept such a vaccine?

NPI COMPLIANCE

1 [*completely disagree*] till 5 [*completely agree*]

1. I am motivated to behave in accordance with the authorities’ recommendations.
2. I have been ready to make changes in my behavior in order not to get infected with the coronavirus.
3. I have been ready to make changes in my behavior in order not to spread the coronavirus.
4. I see no reason to change my behavior despite the corona pandemic.

CONSPIRACY BELIEFS

1 [*completely disagree*] to 5 [*completely agree*]

1. A hidden organization is behind the spread of the coronavirus.
2. Pharmaceutical companies are behind the spread of the coronavirus.
3. Financial interests lie behind the spread of the coronavirus.
4. The coronavirus pandemic is made up.
